# Seed Spillage from Grain Trailers on Road Verges during Oilseed Rape Harvest: An Experimental Survey

**DOI:** 10.1371/journal.pone.0032752

**Published:** 2012-03-09

**Authors:** Diane Bailleul, Sébastien Ollier, Sylvie Huet, Antoine Gardarin, Jane Lecomte

**Affiliations:** 1 Unité Ecologie, Systématique et Evolution, Université Paris-Sud, Orsay, France; 2 Département Mathématiques et Informatique Appliquées, Institut National de la Recherche Agronomique, Jouy-en-Josas, France; New York State Museum, United States of America

## Abstract

**Context:**

Anthropogenic vectors enhance the natural dispersal capacity of plant seeds significantly in terms of quantity and distance. Human-mediated seed dispersal (i.e. anthropochory) greatly increases the dispersal of crop species across agroecosystems. In the case of oilseed rape (OSR), spillage of seeds from grain trailers during harvest has never been quantified.

**Methods:**

Our experimental approach involved establishing 85 seed trap-sites on the road verges of an agricultural area around the grain silo of Selommes (Loir-et-Cher, France). We recorded OSR spillage during harvest and applied a linear model to the data.

**Results:**

The amount of seed spilled was related positively to the area of the OSR fields served by the road, whereas the amount of seed spilled decreased with other variables, such as distance from the trap-site to the verge of the road and to the nearest field.

The distance to the grain silo, through local and regional effects, affected seed loss. Local effects from fields adjacent to the road resulted in a cumulative spillage on one-lane roads. On two-lane roads, spillage was nearly constant whatever the distance to the silo due to a mixture of these local effects and of grain trailers that joined the road from more distant fields.

From the data, we predicted the number of seeds lost from grain trailers on one road verge in the study area. We predicted a total spillage of 2.05×10^6^ seeds (±4.76×10^5^) along the road length, which represented a mean of 404±94 seeds per m^2^.

**Conclusion:**

Containment of OSR seeds will always be challenging. However, seed spillage could be reduced if grain trailers were covered and filled with less seed. Reducing distances travelled between fields and silos could also limit seed loss.

## Introduction

Quantifying dispersal is crucial for understanding the population dynamics and spatial distribution of species [1]. In the case of annual plants, dispersal is governed in general by seed flow [2]. Seed dispersal is often a complex phenomenon that involves different vectors [3], which can be characterised on the basis of their nature (biotic, abiotic or anthropogenic), their temporal action (primary or secondary dispersal), and by the range of distances covered (short or long distance dispersal).

Human-mediated seed dispersal (i.e. anthropochory) influences plant dispersal greatly in areas where human activities are intense [4], [5]. A huge range of anthropogenic vectors disperses seed. For example, seeds can be trapped in mud on footwear [6], [7] or on cars [8], [9], [10], [11], [12], [13]. Cars can disperse seeds in different types of environment as they can travel very long distances [4], [9], [14]. However, car-mediated dispersal is limited by the fact that, in general, cars carry only a small number of seeds. In contrast, in agroecosystems, agricultural machinery can disperse large quantities of seed. Seed can be scattered by mowing machinery [15], [16] and harvesters [17], [18], either *in situ* or while being driven [17], [19]. However, none of the above-mentioned studies, except for Bullock *et al.* [16], have conducted precise measurements of the amounts of seed and dispersal distances, which are required to characterise seed dispersal [1].

Quantifying anthropochory is particularly necessary in agroecosystems, in which seed flow from crops could lead to the establishment of feral populations. Feral populations are crop populations which establish in uncultivated areas, such as road verges. Oilseed rape (OSR; *Brassica napus* L.) is one of the most commonly cultivated crops in the European Union (6.3×10^6^ ha of OSR was cultivated in 2009 [20]), and feral populations of OSR show a strong capacity to persist and retain traits from varieties that are no longer grown [21]. In particular, the spread and persistence of genetically modified (GM) feral populations of OSR are expected to lead to ecological, agricultural, and economic issues, because feral GM plants could constitute reservoirs of transgenes [22]. GM OSR feral populations emerged from seed import spilled during transportation [17], [35]. Moreover the cultivation of GM OSR has been shown to lead to GM feral populations [23] and to GM volunteers, even after GM cultivars are no longer used [24], [25]. GM feral populations are able to exchange genes through pollen flow with other GM feral populations and with GM fields [23], [32], [34]. Seed from OSR could also survive in feral soil seedbanks [26] thus the survival of GM OSR seeds in feral soil seedbanks should as well increase the persistence of transgenes in the environment.

Some studies have attempted to determine the origins of feral OSR populations [27], [28], [29], [30], [31]. With the exception of the mechanism of pod shattering, which is a natural but spatially limited ballistic mode of seed dispersal [27], the origin of feral populations seems to be linked to agricultural machinery and vehicle-mediated seed dispersal. Pivard *et al.* [28] found that feral populations originated mainly from seeds that were scattered from adjacent fields. Crawley & Brown [29] found that feral populations are rarely correlated with previous adjacent field presence. Furthermore, seed banks that originate from scattered seeds are responsible for the persistence of feral populations over time rather than local recruitment [30]. From road verges, seeds can be blown over many metres (maximum distance 21.5 m) by the air displacement caused by vehicle traffic [31].

Surveys of OSR populations in various countries have demonstrated the major role of seed spillage from grain trucks in the spread of OSR which is correlated closely with the transport network. In France, 15% of feral populations were found to originate from the transport of seed by trailers and trucks [28]. In Canada, the presence and persistence of feral populations of GM OSR were correlated with the transportation routes of trucks, for example, between fields and grain silos [17], [32]. Similarly, in Great Britain, seed spillage from trucks was correlated with the persistence of feral OSR populations [29], [33]. In Japan, where no GM OSR is cultivated, feral GM populations were found between ports into which GM seed is imported and oilseed processing facilities [34], [35], [36], [37].

Quantitative estimates of the spillage of OSR seed by grain trailers or trucks have been obtained indirectly through surveys of feral populations of OSR but have been rarely measured directly. To our knowledge, the only study that has quantified the dispersal of OSR seed by vehicles was by von der Lippe and Kowarik (2007a, 2007b, 2008), who conducted seed trapping experiments in tunnels on a motorway between Berlin (Germany) and its agricultural suburbs. They found 6252 viable seeds, of which the most abundant were wheat, cereal rye, and OSR. The type of seed trapped was associated with the direction of traffic flow through the tunnels [38], [39], [40]. In general, few seed trapping studies have been conducted [41] and even fewer studies have quantified long distance human-mediated dispersal [7]. However, the quantification of OSR seed dispersal by grain trailers and trucks is necessary to estimate the risk of long distance seed dispersal from cultivated fields and, consequently, the establishment of feral populations.

To improve knowledge of the dispersal of OSR seed in agroecosystems, we performed an experiment using traps. We laid seed traps on road verges located near OSR fields to measure seed spillage from grain trailers during harvest. We then used statistical models to explain the amount of trapped seed according to several covariables that described the road network, as well as local and landscape elements. We therefore make the five following assumptions:

1. The number of seeds spilled from grain trailers onto the road verge should be related positively to the total area of OSR fields served by the selected road. Indeed, the number of grain trailers needed to transport the harvest from the fields to the grain silo should be proportional to the area of OSR fields harvested.
2. Seed spillage from grain trailers should be greater on two-lane roads (main roads) than on one-lane roads. Drivers of grain trailers heading to the grain silo are expected to use more rapid and direct two-lane roads where possible.
3. The number of seeds spilled from grain trailers should decrease with distance from the nearest OSR field. Grain trailers that have been overfilled with seeds in the field would lose increasingly less seed on the roads as the excess of seed decreases.
4. The amount of seed spilled on road verges should decrease with increasing distance to the edge of the road because more seed is spilled on and near the road.
5. Seed spillage from grain trailers should increase near the grain silo. All grain trailers converge at the grain silo so seed spillage from trailers should also converge at the grain silo.

## Results

**Figure 1:**
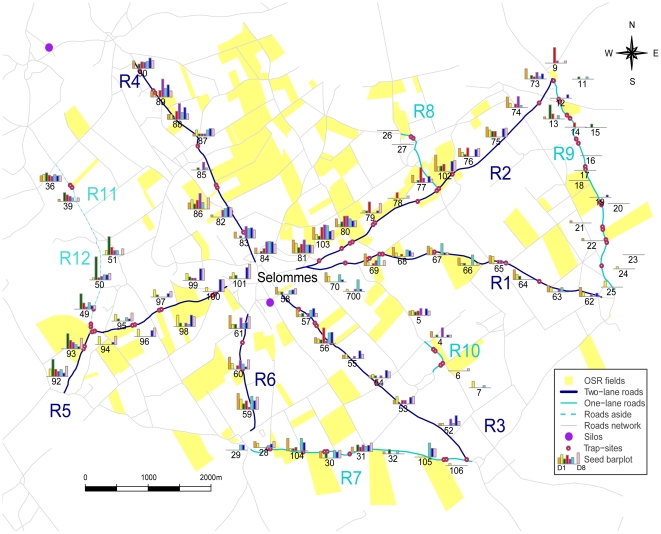
Global map of the Selommes area with daily barplots of OSR seed collected at each trap-site location. Seed amounts are ln(*x*+1) transformed; all barplot axes have the same scale between 0 and 8 (which indicates 0 to 2980 seeds). Each trap-site is associated with a number. Each road is represented by an R-number code. D1 is the first day of the survey and D8 the last day.

We collected a total of 7710 seeds among the 85 trap-sites. We found seeds every day on every road. The amount of seed spilled during the 8-day experiment varied greatly among trap-sites: it ranged from 0 (for example, trap-site 26 on R8, see Figure 1) to 1567 seeds per trap-site (trap-site 50 on R12) with an average of 90.7 seeds per trap-site ±185.1. The spatial variability of seed deposition was higher at the landscape scale than at the road scale. Indeed, the between-roads variability (standard deviation of mean amount of seed per trap-site per road) was 188.9 seeds per trap-site per road and the within-roads variability (mean of standard deviation per trap-site per road) was 115.9 seeds per trap-site per road.

### Spatio-temporal patterns

There was substantial temporal variability with respect to the number of seeds collected at a trap-site. The mean deposition of seed during the 8-day experiment ranged from 0 on R8 to 692.0±794.9 seeds per trap-site on R12. Seven trap-sites on one-lane roads did not collect any seed (R8 and R9 roads, see Figure 1). All the trap-sites on two-lane roads collected seeds during the survey. The maximum number of seeds collected at a trap-site was 1548 seeds on the third day at trap-site 50 on R12 (which collected a total of 1567 seeds during the 8-day survey). Only seven trap-sites collected seeds on every day (trap-sites 36, 59, 80, 81, 103, 87, and 88). However, in general, trap-sites that were close to each other showed the same pattern of daily seed deposition (see, for example, trap-sites 54 and 55 on R4, Figure 1).

Almost all trap-sites collected seeds, regardless of the distance to the nearest OSR field: 92% of the trap-sites at 0 m received seeds, 90% of the trap-sites at 40 m, and 94% of the trap-sites at 400 m.

We did not investigate these spatiotemporal patterns any further because this was not the goal of our study, but they were highly informative in the case of roads R11 and R12, (Figure 1) which received substantial amounts of seed on the third day of the survey. This abundant spillage of seeds could not be related to any of the OSR fields in the study area (data not shown). Grain trailers seemed to be heading along these roads from the south to another grain silo in the north, outside our study area. Therefore, we decided to exclude the data from the five trap-sites on these two roads R11 and R12 from the subsequent statistical analyses.

The stepwise procedure based on the AIC criterium selected the best-fit linear regression model included all the principal variables and the interaction between *Lanes* and *Dist_Silo* (see Table 1). The referential for the qualitative variable *Lanes* was one-lane roads and for **Dist_field,** 0 m. Furthermore, with respect to *Dist_field*, the difference between 0 and 40 m was significant (*P*<0.05), and not significant between 0 and 400 m (*P* = 5.60×10^−2^, data not shown). Data for sites at 40 m and 400 m were thus combined into a new variable: *faraway*.

**Table 1:** Summary of the linear model describing relationships between the natural logarithm of seeds lost +1 and landscape elements.

	Estimate	Standard error	*P*-value
(Intercept)	4.259	0.820	1.81×10^−6^ ***
*Surface_OSR*	9.93×10^−3^	4.23×10^−3^	2.35×10^−2^ *
*Dist_field_faraway*	−0.644	0.236	7.84×10^−3^ **
ln(*Dist_road*)	−0.470	0.183	1.21×10^−2^ *
*Lanes*_2	−0.617	0.800	0.442
*Dist_Silo*	−4.45×10^−4^	1.13×10^−4^	1.97×10^−4^ ***
*Dist_Silo:Lanes*_2	2.95×10^−4^	1.71×10^−4^	8.83×10^−2^ .

. ≤10%;

* ≤5%;

** ≤1%;

*** ≤0.1%.

On a two-lane road, when *Dist_field* is 0 m, the expected response equals (4.259−0.617)+9.93×10^−3^⋅*Surface_OSR*−0.470×ln(*Dist_road*)+(−4.45×10^−4^+2.95×10^−4^)⋅*Dist_Silo* = 3.642+9.93×10^−3^⋅*Surface_OSR*−0.470×ln(*Dist_road*)−1.491×10^−4^ ⋅*Dist_Silo*.

The amount of seed lost on road verges increased with the surface of OSR fields serviced and decreased with distance to the field, distance to the road and distance to the silo (see Figure S1). Indeed, the slope of the linear regression between ln(*Seeds*+1) and *Surface_OSR* was positive (9.86⋅10^−3^, *P*<0.05) and negative with ln(Dist_road) (−0.48, *P*<0.05, see Table 1). When *Dist_field* was *faraway*, the expected response decreased by 0.64 (*P*<0.05). As for the interaction between *Dist_Silo* and *Lanes*, these results were interpreted as followed: the number of lanes was not significant (*P*>0.05) thus *Lanes* had no effect on the origin of the regression line. As for the slope, on a one-lane road, when *Dist_field* was 0 m, the expected response decreased by 4.45×10^−4^ with *Dist_Silo* (*P*<0.05) while on a two-lane road the slope was negative (−1.49×10^−4^, *P* = 0.264, data not shown) but was not significant and thus interpreted as null.

The graphic of standardized residuals versus fitted values does not show any particular structure, confirming thus that the logarithm transformation stabilized well the variance of the observed response. Moran's test for regression residuals was not significant (0.41, *P* = 0.34): residuals of the model were spatially independent according to a neighborhood defined as the inverse of the shortest distance by the road network between two trap-sites and due to the introduction of *Dist_Silo*.

### Predictions

We predicted the number of seeds spilled onto the verge of the two-lane paved road R2 owing to transport by grain trailer. A total spillage of 2.05×10^6^ seeds (1.09×10^6^–3.00×10^6^, 95% confidence interval) were predicted by our model, which represented the harvest of 26 m^2^ of OSR (the average OSR harvest *in situ* is 78849 seeds per m^2^ for an efficiency of 35 quintal per hectare, with 9.5% of harvest lost in situ and 15% humidity [20]) and a mean dispersal of 404 seeds per m^2^±94.

### Emergence Rates

After planting, seeds emerged in nearly every plot (94.4%). The mean rate of emergence for the collected seeds was 80.7%. Some seeds (11.9%) were damaged: half broken or a portion of their tegument detached. Damaged seeds had a lower emergence rate than undamaged seeds (36.8% vs 90.8%).

## Discussion

Spillage of OSR seed from grain trailers during harvest appears to be a very common phenomenon, affecting both main roads to the grain silo and other roads. We showed that seed spillage increased with the surface area of OSR fields served, whereas local elements (e.g. distance from the trap-site to the verge of the road and to the nearest field) decreased the amount of seed spilled at a given site. We found no effect of the number of lanes of the roads alone on the number of seeds lost. We detected an interaction between the number of lanes and the distance to the main silo that influenced the slope but not the origin of the regression line. Seed loss decreased with distance to the main silo on one-lane roads but was constant on two-lane roads. On a road that serviced 66 ha of OSR fields, we predicted the spillage on the verge in the direction of transportation to be nearly two million seeds. In the context of GM crops cultivation in agroecosystems, these results emphasise the need to introduce the landscape complexity in models predicting the presence and persistence of GM OSR feral populations.

To our knowledge, there have been no previous studies on the amount of crop seed lost on road verges from grain trailers. Few studies have quantified seed dispersal by vehicles. For example, a study of car-mediated dispersal of seeds stuck into mud attached to vehicles was conducted on Flemish roads [14], in which the car-borne flora included seeds quite similar to OSR seeds (small, light seeds able to establish a persistent seedbank). The results predicted that an average of 2.6 million cars disperse 7.8 million seeds each year on Flemish roads. In contrast, our prediction of OSR seed spillage by grain trailers was much higher: nearly 2 million seeds lost in only 8 days on a single road of 5 km. Von der Lippe & Kowarik [39] laid traps in tunnels in suburban Berlin to collect seed spilled from vehicles. They estimated an annual seed loss that ranged from 2 to 67 seeds per m^2^ and 0.4 to 14.4 seeds per trap, whereas we obtained an average of 11 seeds per trap-site per day with smaller traps, so approximately 66 seeds per m^2^ per day. Thus, if comparison is possible between these three studies, grain trailers appear to contribute more to seed dispersal than cars and seed dispersal seems to be more abundant in agricultural areas than in suburban areas.

On verges, seeds were spilled mostly near the road, regardless of the type of road, despite our preliminary hypothesis. No previous study on dispersal is dealing with road type and grain trailers or trucks to our knowledge either. From what we observed on field, the absence of interaction between the distance to the road and the type of road on the amount of seed collected could be explained by different styles of driving on the different types of road. Seed losses through cracks in the back doors are presumed negligible and must occur mostly from the top of the trailer, which was rarely covered in our study area. Wind turbulence across the top increased with speed and so, seeds spilled. On narrow one-lane roads, grain trailers were driving slower and nearer to the verge, whereas on two-lane roads, grain trailers drove faster and in the middle of the road (pers. obs.) but also seemed to allow seeds to disperse further.

As for the relationship between the number of seeds spilled and the distance to the field, our hypothesis was confirmed by the statistical analysis. Less seed was spilled at trap-sites located 40 m and 400 m from the field than at field borders. Indeed, grain trailers are commonly overfilled with seeds. The excess seed seems to be usually spilled within the first 10 m that the grain trailers drove on the road. When the excess had been lost and the seeds had been compacted in the trailer, spillage decreased. Given that the amount of seeds lost decreased with distance from the field, we presumed that at a certain distance from the field, spillage should cease.

Distance to the silo was a predominant variable that interacted with the number of lanes and the geography of the area. The result that seed spillage decreased with the distance to the silo on one-lane roads was expected. However, seed spillage on two-lane roads was constant with the distance to the silo. Few grain trailers travelled on one-lane roads, and for short distances because the grain silo was at the centre of the area and one-lane roads were in general peripheral. Therefore, seed spillage on one-lane roads should be related only to local fields. Two-lane roads always led to the grain silo and thus received also seed spillage from all the neighbouring fields from other roads. This led to a non-structured and more heterogeneous cumulative pattern than on one-lane roads.

The unique event that spilled 1500 seeds (i.e. 20% of the total number of seeds collected during the eight days) at a single trap-site could not be correlated with any OSR field in the study area. The roads that were affected by the high levels of spillage on day 3 of the study period (R11 and R12) were on the edge of the study area and the grain trailers involved in the spillage probably originated from a farm outside the study area. This massive deposition of seeds also demonstrated that grain trailers can disperse seeds over distances greater than 400 m. Despite the large amounts of seed spilled and the high overall emergence rate under greenhouse conditions (80.7%), no feral populations were observed on road R12 in 2011, or during the previous 10 years. The road R12 is bordered by forest and low availability of light could explain the absence of feral OSR. Indeed, the deposition of seed is not necessarily related to adult recruitment, because the environmental conditions where seeds fall play a major role [42].

We showed that grain trailers frequently dispersed seeds up to a distance of 400 m. This is the farthest dispersal distance for OSR seed ever quantified. Environmental conditions such as traffic [31] and wind velocity favoured secondary dispersal (shown for wind-dispersed seeds [43], [44]). Seed dispersal under these conditions could increase dispersal distances and trapped amounts of seeds. Inversely, owing to our experimental design, dispersal distances might have been highly underestimated because seeds could be dispersed further if not trapped [45].

### Synthesis and Perspectives

Despite its limitations, this study is the first to quantify the spillage of OSR seed from grain trailers, highlight the importance of this phenomenon, and explain seed loss on the basis of local and landscape elements.

According to Pivard *et al.* [28], feral populations of OSR originate from the immigration of seed from fields (35–40%), from local recruitment (10%), from persistent seed banks (35–40%), and from seed transportation (15%). From all the potential sources of feral populations, spillage from grain trailers is the only one which can disperse OSR seeds farther than tens of metres. Although emergence rates of collected seeds in the field were high, conditions *in situ* will determine rates of seed germination, survival or secondary dormancy. In a context where GM plants are cultivated, GM OSR seeds will be dispersed farther than expected by actual estimations. Road verges management should take into account these dispersal distances. Although it might be impossible to eliminate the spillage of small seeds, such as OSR, during transportation, spillage from grain trailers could be reduced by the introduction of appropriate policies. For example, covering the top of grain trailers, not overfilling trailers, or reducing the distances travelled between fields and silos could limit the loss of seed.

In general, seed spillage from grain trailers is not considered or is underestimated in models for seed dispersal and the persistence of feral populations of GM OSR [46], [47]. To enable seed dispersal by grain trailers to be integrated into mechanistic models, further study of the phenomenon is required. To perform genotypic analyses, we collected mature leaves from the germinated seeds from the traps and from OSR fields cultivated in 2010. By determining genotypes in specific fields, we would be able to assign trapped seeds to particular sources and thus evaluate realistic dispersal distances. Our study has highlighted the role of the surface area of fields and transportation to grain silos in seed dispersal by grain trailers. Local and landscape elements and rare events of massive seed deposition should also be integrated into models evaluating GM crops escape and their persistence.

## Materials and Methods

### Study Area

The study area was a typical open-field agricultural landscape of 41 km^2^ centred on the village of Selommes, Loir-et-Cher, France (47°45′24″N; 1°11′34″E) which contains a grain silo where most local farmers take their harvested grains (see Figure 1). Local farmers use grain trailers to transport their seeds from fields to silo. In 2010, 185 field plots of winter OSR were cultivated in this area over a total of 684 ha (16.7% of the area). Feral populations of OSR were found on 10–14% of the road verges each year over the last ten years [30]. Fields, populations, roads, and many landscape elements were recorded and mapped. Roads were categorised as paths (42% of roads) and one-lane (29%) or two-lane (29%) paved roads.

No specific permission was required for this study as french roads are public areas. Road verges are not privately-owned or protected. The DDE (Direction Départementale de l'Equipement, in charge of road verges management), town mayors and farmers were informed about the study before the experiment.

### Experimental Design

We focused on the study of paved roads more than 600 m long as this length was sufficient to put several traps at various distances from fields. We used as reference a survey from local farmers about grain trailer trips between fields and the main silo (survey data, [20]). Based on this survey, we computed the area of adjacent OSR fields along each road. Then, the 21 roads were classified on the basis of the area of the OSR fields that they served (four increasing categories of field areas) and the number of lanes (one or two). We randomly selected two roads when possible in each of the eight categories. Twelve roads were then selected, half in each lane category. The regular presence of feral populations along almost all the possible roads was a rough indication of seed loss from grain trailers.

Traps were placed at the beginning of the OSR harvest season in 2010. Over the subsequent eight days, all traps were checked daily (except for the final record, which was taken after a gap of two days) and emptied of seeds. At the end of the experiment, all the surrounding fields have been harvested. Grain trailers are the only seed transportation vehicles circulating during harvest.

Each trap consisted of two rectangular plastic saucers (172×425 mm) that were positioned side by side to form a 1-m long trap, parallel to the road, the nearest as possible to the road, and secured to the ground with two camping pegs each. A fine layer of cotton wool was placed in the base of each trap to prevent seeds from ricocheting out. Vegetation around the traps was cut back before installation to prevent it from disturbing the fall of the seeds into the traps [48]. The two saucers were collected together because considered as single trap.

### Data collection

We pooled the number of seeds collected in each trap over the eight days. Thus, the dependent variable in our model was the total number of seeds.

To explain the total number of seeds (*Seeds*, quantitative variable) found in the traps over the eight days, five explanatory variables were defined (see Table 2):

1. *Surface_OSR*: the total area of OSR fields serviced by the selected road. We calculated these areas according to the survey of trailer trips.
2. *Lanes*: the number of lanes of the selected road. Two-lanes roads are generally straight and heading to the grain silo whereas one-lane roads are making path between two-lanes roads or fields and two-lanes roads.
3. *Dist_field*: distance between each trap-site and the nearest OSR field. We placed traps at 0 m, 40 m, and 400 m from OSR field, along the side of the road that was directed towards the main grain silo. This gave a total of 85 trap-site locations: 37 at 0 m, 30 at 40 m, and 18 at 400 m. In addition to grain trailers, combine harvesters also scatter crop seed in fields [5], [49] and on the borders of fields [19]. To measure only spillage from grain trailers, trap-sites located at 0 m from fields were put adjacent to the fields, with a gap of about 5 m.
4. *Dist_road*: distance from the edge of the road to the trap-site on the road verge. Even if traps were placed the nearest as possible to the road, theirs positions differ according to the road or the soil nature. We also tested the interaction between this variable and *Lanes*. Given that one-lane roads are narrow and two-lane roads are straight and wide, grain trailers are expected to drive differently on these roads, which should affect the pattern of seed spillage on road verges.
5. *Dist_Silo*: distance between each trap-site and the main Silo of Selommes. As each trap was mapped, the distance to the main silo was computed through the road network. We tested also the interaction between *Lanes* and *Dist_Silo*.

**Table 2:** The five explanatory variables for the number of seeds recorded in trap-sites.

	Modalities	Description
*Surface_OSR* (ha)	From 2.8 to 147.5	Total area of all OSR fields adjacent to the road and connected to the road by network^1^
*Lanes*	1 or 2	Number of lanes on the road
*Dist_field* (in m)	0, 40 or 400 then 0 or faraway	Distance between the trap-site and the nearest OSR field
*Dist_road* (in m)	from 2.4×10^−2^ to 1.7^2^	Distance between trap-sites and the edge of the road
*Dist_Silo* (in m)	From 1073 to 8848	Distance between the trap-site and the grain silo at Selommes^3^

1 Based on a farmer survey concerning trips between fields and silos (from CETIOM data [20], 1999).

2 Only four trap-sites were placed at more than 1 m.

3 Calculated along a given road from georeferenced data with R software.

### Statistical Modelling

The relation between the observed amounts of seeds and the covariates is modeled via a linear model where the dependent variable is ln(*Seeds*+1). The model included all the variables (see Table 1) and the interactions between *Lanes* and *Dist_Silo*, and between *Lanes* and *Dist_road*. The dependent variable *Seeds* was ln-transformed to stabilise the variance to fulfil the assumptions of the linear model. *Dist_road* was also considered through its logarithm as ln(*Dist_road*). We chose the model that fit our data best according to Akaike's information criteria (AIC) and analysis of residuals and partial residuals of the explanatory variables.

### Model analysis

To evaluate the spillage of OSR seeds from grain trailers along road verges, predictions were made using the best linear model identified previously. This model was used to predict the total number of seeds that dispersed along the verge in the direction of traffic flow to the silo on a given road; road R2 was selected (Figure 1). R2 is 5078 m long and services 28 OSR fields with a total area of 66.4 ha. Road verges were approximately one metre wide. To make predictions on a squared metre at a time, as trap-sites were one metre long and 17.2 cm wide, we predicted seed amounts at 6 different distances from the edge of the road to obtain a metre wide, for every metre of the R2 road.

The predict function calculated the predicted amounts of seed and we approximated a prediction interval at the road scale.

### Emergence Rate of Collected Seeds

To test for seed viability and identify OSR, we germinated every seed collected in rich soil under greenhouse conditions. All the seeds collected on one day from one trap-site were grown in the same plot. For one sample that comprised more than 1500 seeds, we only planted 140 seeds. Given that the emergence rate for this plot was 100%, we did not test any more seed from this sample. We surveyed the seedlings until their first leaves appeared.

We used R software [50] for all the analysis.

## Supporting Information

Figure S1 **Representations of the amount of seed (ln(** ***Seeds*** **+1)) as a function of the variables of the study.**
(TIF)
