# Supplementary figures and images for: Seed Spillage from Grain Trailers on Road Verges during Oilseed Rape Harvest: An Experimental Survey
Source: PLoS One. 2012 Mar 9;7(3):e32752. doi: 10.1371/journal.pone.0032752 (PMC3302880; doi:10.1371/journal.pone.0032752)

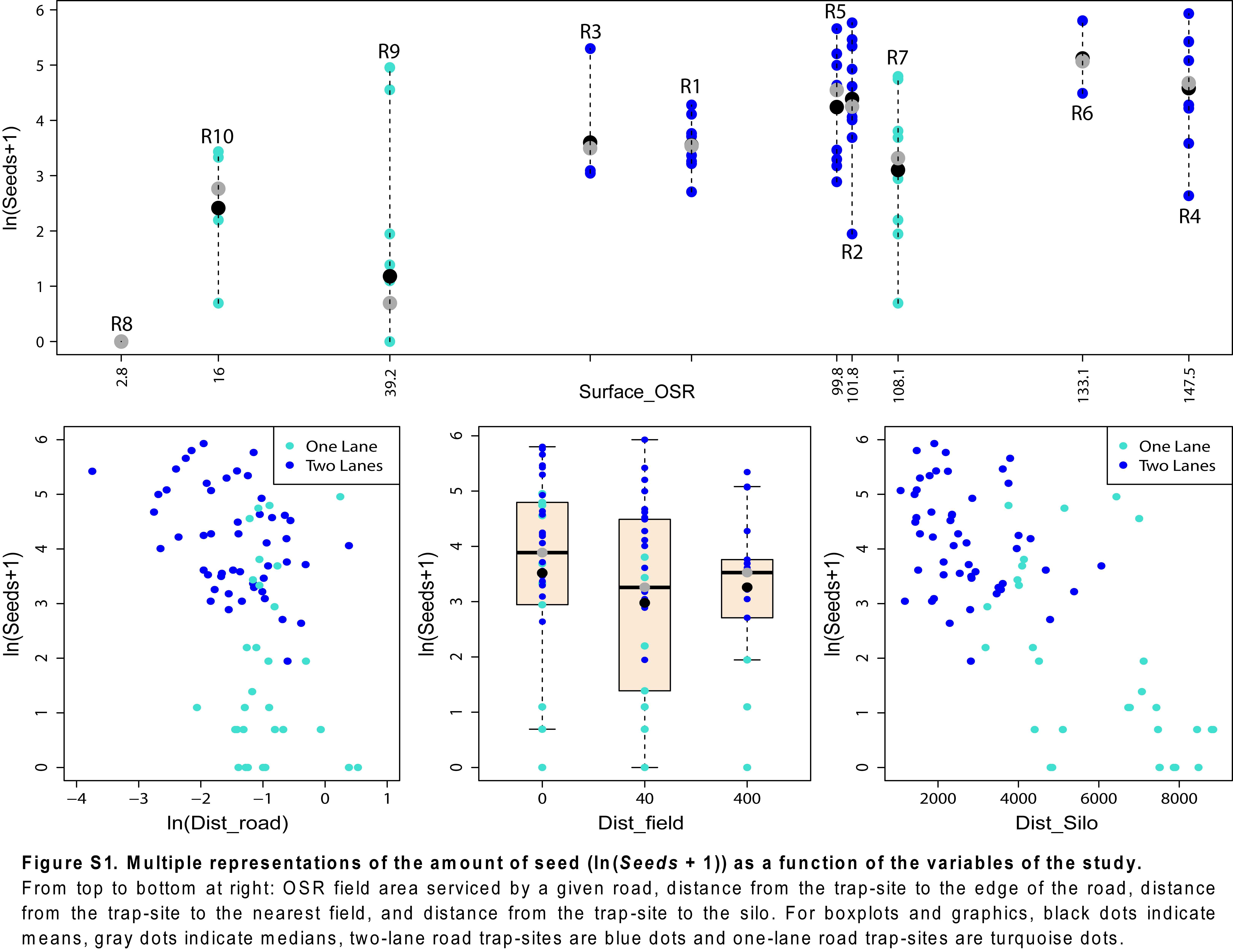

Supplement: Figure S1 — Representations of the amount of seed (ln( Seeds +1)) as a function of the variables of the study. (TIF) [file pone.0032752.s001.tif]
